# Functional Impairments and Dementia Incidence in the English Longitudinal Study of Ageing

**DOI:** 10.1093/geroni/igaa057.2293

**Published:** 2020-12-16

**Authors:** Dorina Cadar, David Llewellyn, G David Batty, Andrew Steptoe

**Affiliations:** 1 University College London, London, England, United Kingdom; 2 University of Exeter Medical School, Exeter, England, United Kingdom

## Abstract

Functional disability might be related to an increased risk of dementia or could represent a prodromal stage. We examined the occurrence of functional impairments over eight years follow-up and their association with dementia incidence in 1,666 participants aged 65+ from the English Longitudinal Study of Ageing. Growth models with distal outcome were used to examine whether different trajectories of functional abilities (activities of daily living (ADL); and instrumental activities of daily living (IADL)) between 2002/03 and 2010/11, were associated with dementia incidence four years later (2014/15). Participants with an increasing number of functional impairments (Class III), were more likely to be classified with subsequent dementia compared with those with no impairments (Class I). An increased risk was also observed for individuals with raised levels of impairments (Class II). We found IADLs more sensitive than ADLs, and this may imply a more comprehensive ascertainment during the prodromal stage of dementia.

